# 1479. Analysis of Depression in People Living with Human Immunodeficiency Virus (HIV) During the Coronavirus Disease 2019 Pandemic in 4 African Countries

**DOI:** 10.1093/ofid/ofad500.1315

**Published:** 2023-11-27

**Authors:** Paul Adjei, Nicole Dear, Allahna Esber, Hannah Kibuuka, Jonah Maswai, John Owuoth, Valentine Sing’oei, Emmanuel Bahemana, Trevor A Crowell, Julie A Ake

**Affiliations:** Walter Reed Army Institute of Research, Silver Spring, Maryland; Henry M. Jackson Foundation for the Advancement of Military Medicine, Bethesda, Maryland; Henry M. Jackson Foundation for the Advancement of Military Medicine, Bethesda, Maryland; Makerere University Walter Reed Project, Kampala, Kampala, Uganda; U.S. Army Medical Research Directorate - Africa, Kericho, Kenya, Kericho, Western, Kenya; Henry M. Jackson Medical Research International, Kisumu, Western, Kenya; Henry M. Jackson Medical Research International, Kisumu, Western, Kenya; Henry Jackson Foundation for the Advancement of Military Medicine, Mbeya, Mbeya, Tanzania; Henry M. Jackson Foundation for the Advancement of Military Medicine, Bethesda, Maryland; Walter Reed Army Institute of Research, Silver Spring, Maryland

## Abstract

**Background:**

We previously demonstrated higher odds of depression in PLWH during COVID-19 pandemic in our African Cohort Study (AFRICOS) between 07May2020 and 01Dec**2021**.^1^

This analysis updates the previous and estimates odds of depressive symptoms through 01Dec**2022**.

Hypothesis: If the pandemic was truly associated with depressive symptoms in PLWH then odds of depressive symptoms will continue the downward trend identified in later timepoints of our first analysis.

**Figure:**
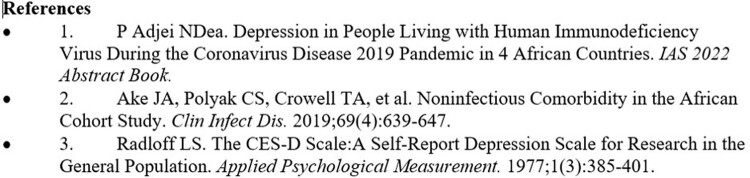

**Methods:**

AFRICOS: Ongoing prospective cohort study enrolling PLWH at 12 clinics in Kenya, Uganda, Tanzania, Nigeria.^2^

Longitudinal evaluations every 6 months include physical evaluation, medical record abstraction, extensive questionnaires including CES-D; depression defined by score >16.^3^

We compared odds of depression in preCOVID-19 period (1 Jan 2019 to 19 Mar 2020) with five timepoints in COVID-19 era (7 May 2020 to 1 Dec 2022).

Logistic regression with generalized estimating equations for OR and 95% CI comparing depression before and during pandemic.

Models *a priori* adjusted for sex, age, and site.

**Results:**

2,038 AFRICOS participants with complete cases and visits before and during COVID-19 periods included.

Median age at first visit included: 43 years (IQR 36-51)

1,181 (57.9%) were female

Depression varied significantly by: sex, participants by site, marital status, ART adherence at first timepoint included (**Table 1**).

60 (2.9%) participants had CES-D score suggestive of depression at first visit included.

Compared with pre-pandemic:

PLWH had higher odds of reporting depressive symptoms throughout pandemic. After adjusting for age, sex, and site, PLWH had higher odds of reporting depressive symptoms only in second, third, fourth parts of pandemic period, aORs: 1.62 (95% CI 1.13 – 2.31), 1.72 (95% CI 1.27-2.31), 1.41 (1.01 – 1.96) respectively (**Table 2, Figure**).

**Figure:**
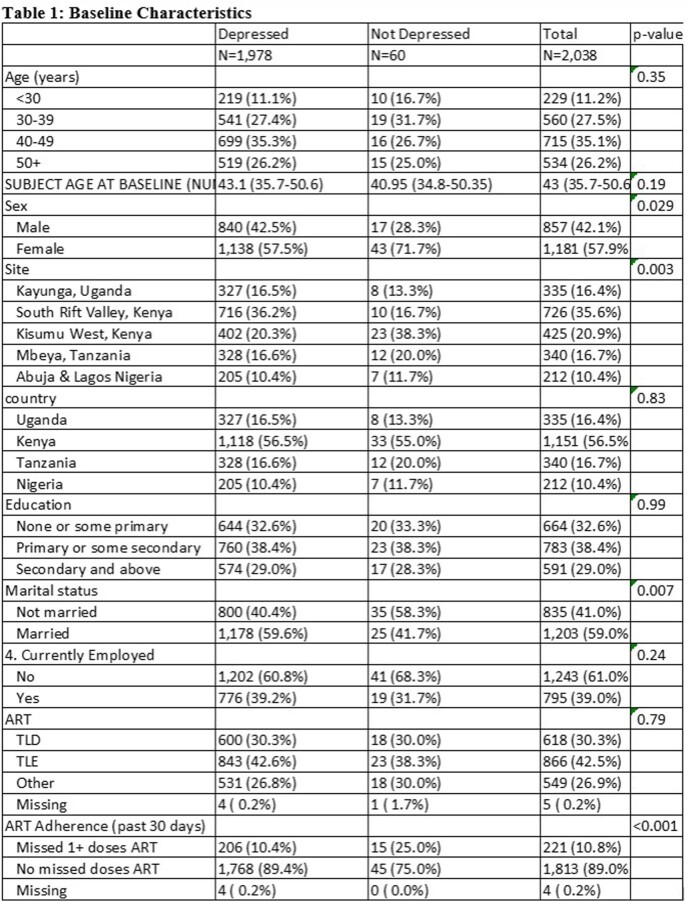

**Figure:**
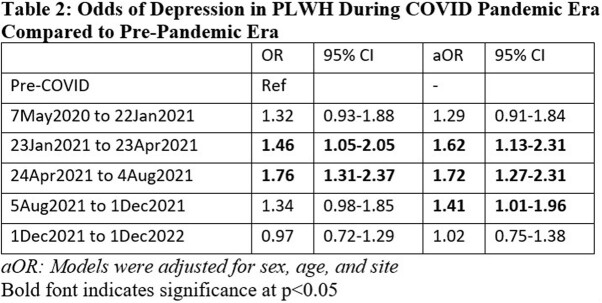

**Figure:**
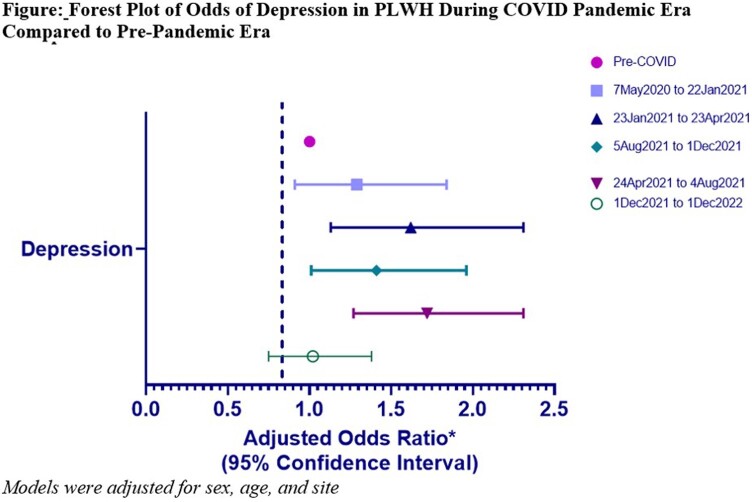

**Conclusion:**

COVID-19 pandemic is associated with higher odds of depressive symptoms in PLWH in AFRICOS.

Odds of depression increased as pandemic progressed, peaking in third period and decreasing sequentially in fourth, fifth periods to confirm our hypothesis.

Given the strong association between depression and HIV outcomes, aggressive public health mental health response to pandemics may improve HIV clinical outcomes.

**Disclosures:**

**All Authors**: No reported disclosures

